# Depression and its associated factors: perceived stress, social support, substance use and related sociodemographic risk factors in medical school residents in Nairobi, Kenya

**DOI:** 10.1186/s12888-021-03439-0

**Published:** 2021-09-08

**Authors:** Sayed Shah Nur Hussein Shah, Ahmed Laving, Violet Caroline Okech-Helu, Manasi Kumar

**Affiliations:** 1 School of Medicine, University of Nairobi, Nairobi, Kenya; 2 Department of Paediatrics and Child Health, Kenyatta National Hospital, University of Nairobi, Nairobi, Kenya; 3 Department of Mental Health, Kenyatta National Hospital, Nairobi, Kenya; 4 Department of Psychiatry, University of Nairobi, Nairobi, Kenya

**Keywords:** Depression, Kenya, Africa, Medical residents, Perceived stress, Social support, Substance abuse, Educational environment, Doctors, Mental health

## Abstract

**Background:**

Little data exists regarding depression and its associated factors in medical residents and doctors in Sub-Saharan Africa. Residents are at high risk of developing depression owing to the stressful nature of their medical practice and academic training. Depression in medical residents leads to decreased clinical efficiency, and poor academic performance; it can also lead to substance abuse and suicide. Our primary aim was to measure depression prevalence among medical residents in Kenya’s largest national teaching and referral hospital. Secondary aims were to describe how depression was associated with perceived stress, perceived social support, substance use, and educational environment.

**Methods:**

We sampled 338 residents belonging to 8 different specialties using self administered questionnaires in this cross-sectional survey between October 2019 and February 2020. Questionnaires included: sociodemographics, the Centres for Epidemiology Depression Scale - Revised, Perceived Stress Scale, Multidimensional Scale of Perceived Social Support, Alcohol, Smoking and Substance Involvement Screening Test, and Postgraduate Hospital Educational Environment Measure. Bivariate and multivariate linear regression were used to assess for risk factors for depression.

**Results:**

Mean participant age was 31.8 years and 53.4% were males. Most residents (70.4%) reported no to mild depressive symptoms, 12.7% had moderate, and 16.9% had severe depressive symptoms. Most residents had high social support (71.8%) and moderate stress (61.6%). The educational environment was rated as more positive than negative by 46.3% of residents. Bivariate analyses revealed significant correlations between depressive symptoms, perceived stress, substance use, perceived social support, and educational environment. Multivariate analysis showed that depression was strongly associated with: fewer hours of sleep (β = − 0.683, *p = 0.002*), high perceived stress (β = 0.709, *p < 0.001*) and low perceived social support (β = − 2.19, *p < 0.001*).

**Conclusions:**

Only 30% of medical residents in our study had moderate and severe depressive symptoms. Most residents in our study reported high levels of social support, and moderate levels of stress. Though their overall appraisal of medical residency experience was positive, mental health support and self-care skills in the training of medical professionals needs prioritization.

**Supplementary Information:**

The online version contains supplementary material available at 10.1186/s12888-021-03439-0.

## Background

As doctors undergoing training it is important for residents to function optimally as they play a key role in a healthcare system. Depression in residents can lead to an increase in medication errors made [1]. Errors made in medical practice can have serious consequences for patients and their families. Negligent care causes 28% of adverse events, 13.6% of adverse events result in patient death. Adverse events due to negligent care are also more likely to result in serious disability to patients [2, 3].

Depressive disorders are characterized by ‘*the presence of sad, empty, or irritable mood, accompanied by somatic and cognitive changes that significantly affect the individual’s capacity to function*’ [4]. Some of the symptoms experienced due to depression include: depressed mood, loss of pleasure or interest, weight loss, insomnia or hypersomnia, psychomotor agitation or retardation, loss of energy/fatigue, guilty feelings, feeling worthless, difficulty concentrating, and suicidal ideation [4].

In 2016, depressive disorders accounted for 2% of global disability adjusted life years (DALYs) varying by region, age and gender with more than 44 million DALYs attributable to depressive disorders [5]. In 2016 in Kenya, 221,500 DALYs were due to depressive disorders [5]. A study in 2002 at the height of the HIV/AIDS pandemic in Kenya found that 48% out of 50 residents in internal medicine, general surgery, paediatrics, and obstetrics & gynaecology met the criteria for major depressive disorder [6]. That is slightly higher than the 42% found in adult non-psychiatric patients and the 41.3% found in university students in Kenya [7, 8]. Despite these studies, risk factors for depression in residents in Kenya are unknown, as well as the prevalence of depression under current conditions. Detailed analyses on the relationships between stress, sociodemographics and depression in this population are lacking. Other important associated factors of depression such as social support, substance use, and educational environment have never been studied in this population.

Depression and mental health problems can be seen in professionals with high stress and pressured jobs. Medical residents are one such category. The development of depression follows a diathesis-stress model, relying on external stressors, the individual’s response to those stressors, as well as the individual’s genetics [9]. Residents experience a large number of stressors and are therefore at high risk of suffering from depression.

The stressors during residency can be divided into three categories: *situational, personal, and professional* [10]. *Situational stressors* include factors such as lack of sleep, many responsibilities (administrative and clerical), lack of support from health professionals, long hours, heavy workloads, many patients, and less than optimal learning conditions [10]. *Personal stressors* include factors such as family that are a source of conflict and stress, limited free time to relax, financial difficulties, psychosocial concerns, and inadequate coping skills [10]. *Professional stressors* include difficult patients and cases, responsibility for patients, supervision of junior residents, information overload, and career planning [10].

Factors such as stress, substance use, social support, and the educational environment each have an effect on the wellbeing of residents and their patients.

A finding from Kenya was that 60.9% of residents had moderate and severe stress [11]. Stress is associated with a higher frequency of malpractice claims, interventions to reduce stress have resulted in fewer medication errors [12]. Stress in residents is also associated with higher burnout and reduced empathy towards patients [13]. Doctors that are more stressed think about leaving their specialty more often than those that are less stressed [14]. Doctors are more likely to commit suicide than the general public [15] with some studies reporting the suicide rate for female doctors being 3 times as expected based on population values, of these female suicides, 29% were undergoing training [16]. Therefore, high stress among doctors can be harmful to both the doctors and the patients that they treat.

Doctors’ health behaviour affects both how they counsel patients on prevention, as well as patients’ attitudes and motivation to make lifestyle changes [17–19]. If doctors abuse substances it may affect their ability to interact with patients not to mention the negative effects substances such as tobacco, narcotics, and alcohol have on the body.

Social support positively influences psychological health [20, 21]. Residents that are satisfied with their current internship tend to report higher levels of social support [22]. Feeling supported by colleagues and management is associated with improved psychological welfare, less avoidance, and less defensive medical practice among doctors [23]. Poor social support can result in emotional exhaustion [24], is related to physical disease, mental disease, and is also linked to morbidity and heart disease [25]. Social and emotional support may also reduce the effects of stressful events, and yield in increased coping with diseases and risk factors such as smoking, and drinking [25, 26]. Doctors are more sensitive than other healthcare workers to low support from their colleagues and to having limited opportunities to control their job [24, 27]. Strong support structures can also compensate for difficult working hours, and for negative stressors among doctors [28]. A strong social support system is therefore very critical for medical residents to thrive in an otherwise demanding and challenging work and training environment.

A positive perception of one’s learning environment correlates to lower levels of burnout [29]. Higher perceptions of role autonomy, and teaching amongst doctors is associated with greater satisfaction in their internship [22]. Poorer ratings of the educational environment are associated with a higher frequency and intensity of stress among doctors [22]. Residents in training with mental illness find it harder to study for postgraduate exams and perform their duties. Some are also afraid of appearing weak and unstable if found experiencing psychological stress resulting in them experiencing job insecurity and avoiding seeking medical help [30].

**Fig. 1:**
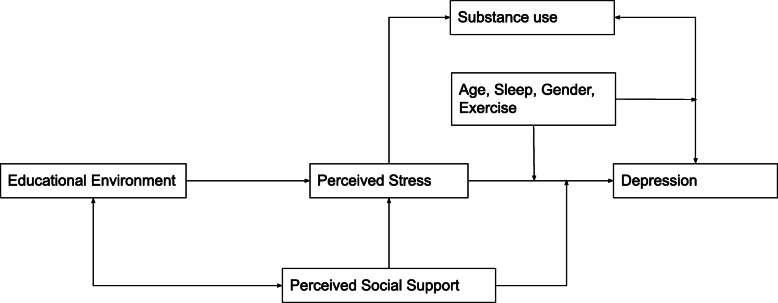
Conceptual Framework for Depression, Stress, Social Support, Substance Use, Educational Environment, and Sociodemographics

We used a conceptual framework (see, Fig. 1) to better understand the complex relationships between depression and all the variables discussed above. To summarise, perceived stress mediates the effects of educational environment and perceived social support on depression. Depression is directly related to perceived stress and substance use with sociodemographic factors such as age, gender, and sleep moderating the effects on depression.

It is therefore important to investigate depression, its prevalence, and the factors associated with it among residents especially in a region like this where scanty data exist for such a population and psychosocial arrangements for frontline workers and medical professionals are scarce.

## Methods

Our study used a cross-sectional design where a subset of mental health assessment tools were used. Our primary aim was to determine the prevalence of depression among medical residents in the largest teaching hospital in Kenya. Our secondary aims were to understand how depression was associated with factors such as perceived stress, perceived social support, substance use, sociodemographics (an additional file shows the sociodemographic questionnaire in more detail (see Additional file 1)), and the educational environment of residency training. The sociodemographic questionnaire included questions about various aspects of residents’ lifestyles. For example, if they participated in any hobbies (we phrased this by asking residents if they carried out any activities for fun/enjoyment in their free time).

### Sample, setting and tools

Sample size was calculated using the formula of Fisher et al. [31]. We required 338 residents according to the sample size calculation, and successfully sampled 338 residents out of 624 in a large teaching hospital in Nairobi, Kenya. The hospital is one of the largest public referral hospitals in the country and is located in an urban setting with high numbers of patients referred from around the country.

The medical residency program in Kenya involves qualified doctors enrolling in postgraduate programs in order to attain a specialisation in a particular field such as psychiatry, general surgery, paediatrics, etc. Residency programs are typically divided into several Departments and these departments might vary in their size and focus from one institution to another. The residency programs have varying durations ranging from 3 to 6 years depending on the specialty. During training, residents work in the hospital and are responsible for all aspects of patient care, with increasing responsibility as they progress through the program. They are overseen by consultants (qualified specialists), who are in charge of patient management, and who also are part of teaching faculty in the University or University hospital structure. In addition to their clinical responsibilities, residents have to participate in academic activities such as group discussions, grand rounds, exams, and dissertations. The courses are taught entirely in English, meaning that residents are proficient in English. Therefore the questionnaires we used were all in their original English forms and did not need translation.

Residents from 8 different specialties in all years of study (first to sixth year) were sampled using proportionate random sampling: 77 from obstetrics and gynaecology, 59 from internal medicine, 51 from paediatrics, 47 from general surgery, 38 from anaesthesia, 29 from psychiatry, 28 from Ear Nose Throat (ENT) surgery, and 9 from cardiothoracic surgery. We selected the above-mentioned specialties in order to obtain a mix of different lifestyles and demands experienced by each, we included procedural specialties such as surgery and non-procedural ones such as psychiatry. Due to budget constraints, we were unable to sample every specialty in the hospital and therefore settled on 8 specialties. The hospital has a total of 870 residents, with a total of 624 belonging to the departments of interest to our study: 100 belonging to internal medicine, 137 to anesthesia, 130 to obstetrics/gynecology, 91 to paediatrics, 40 to psychiatry, 67 to general surgery, 14 to cardiothoracic surgery, and 45 to ENT surgery. A total of 23 residents either refused to participate (2 residents) or refused to submit their questionnaires (21 residents). Residents were selected at random; they were approached by research assistants in their respective wards, clinical teaching rooms, lecture theatres, and within each department. Residents were asked to confirm the specialty and year of study that they belonged to before being given self-administered questionnaires to complete. The residents were allowed to complete the questionnaires at their convenience if they requested to do so and those questionnaires were later collected by the research assistants. Research assistants ensured that all residents were able to complete their questionnaires in private. Given the sensitive nature of the questionnaires, we did not use class lists, nor did we record the names of residents that filled in questionnaires. This was communicated to the residents as well, in order to encourage participation and truthful responses from them. Residents were only allowed to fill in one set of questionnaires.

Data were collected between October 2019 and February 2020, after ethical approval was given by the hospital and university Institutional Review Board (Kenyatta National Hospital –University of Nairobi Ethics and Research Committee, reference UP831/12/2018). All methods including participant recruitment and consent taking, data collection, data handling, and analysis were performed in accordance with relevant guidelines and regulations including the Declaration of Helsinki. The questionnaires were disseminated and collected by two research assistants along with the first author who were trained in administering the tools, human subjects protection, ethical data collection principles and psychological first aid by the co-authors. Written informed consent was obtained and responses were anonymised.

### Depression measure

We used the Centers for Epidemiologic Studies Depression Scale Revised (CESD-R) [32]. It is a 20 items questionnaire that measures depression in 9 different symptom groups (sadness, loss of interest, appetite, sleep, concentration, feelings of worthlessness, fatigue, agitation, and suicidal ideation) outlined by the Diagnostic and Statistical Manual of Mental Disorders, 5th Edition (DSM 5) [4]. It utilises a five-point Likert scale, total scores range from 0 to 60 with higher scores indicating more numerous/severe depressive symptoms. A score greater than 15 indicates a person at risk for clinical depression. The scale has been found to be valid and has shown good reliability and internal consistency (Cronbach’s α = 0.928) for screening for depression in the general population [32, 33]. It has similar characteristics to its predecessor the Centers for Epidemiologic Studies Depression Scale (CESD) [34], and its scoring [35] has been adjusted to match that of the CESD. We used the scoring system used for the CESD: 0 to 15 mild/no depression, 16 to 23 moderate, and 24 to 60 severe. We used the ‘CESD’ scoring system in order to allow us to compare our results to previous studies that used the CESD instead of the CESD-R whose scoring system is not widely used. The CESD has been used in Kenya with good reliability and validity [8].

### Perceived stress measure

We used Cohen’s Perceived Stress Scale (PSS) [36]. It is a 10-items scale that measures how stressful an individual perceives situations in their life to be [36]. It uses a 5-point Likert scale with total scores ranging from 0 to 40; higher total scores indicate higher levels of stress. It has been shown to be valid and reliable in several studies with a Cronbach’s α of 0.85 [36–38]. We used the following classification of PSS scores: 0 to 13 mild stress, 14 to 26 moderate stress, and 27 to 40 severe stress [39]. PSS too has been used in Kenya on different populations with good reliability and validity [40].

### Perceived social support measure

Perceived social support was measured using the Multidimensional Scale of Perceived Social Support (MSPSS) [41]. The MSPSS is a 12 item score that assesses perceived social support from family, friends, and a significant other. It uses a 7-point Likert scale, with higher scores indicating higher levels of social support. It has good validity, reliability (Cronbach’s α = 0.90) and test-retest scores [41]. We used the categorisation outlined in the MSPSS where mean scores ranging from 1 to 2.9 are low support, 3 to 5 are moderate support, and 5.1 to 7 are high support [41]. The MSPSS has been used in Kenya before with good reliability [42].

### Hospital educational environment measure

The Postgraduate Hospital Educational Environment Measure (PHEEM) [43] was used to assess the educational environment. It is a 40 items questionnaire divided into 3 sections that assess perceived role autonomy, perceived teaching support, and perceived social support. It has been shown to be valid and reliable (Cronbach’s α = 0.93) [43–45]. The scoring categorisation outlined in the development of the PHEEM was used to classify scores [43]. This was the first time PHEEM has been used in Kenya.

### Substance use measure

Substance use was assessed using the Alcohol, Smoking and Substance Involvement Screening Test (ASSIST) [46]. It is a tool developed by the World Health Organisation (WHO) to ‘detect psychoactive substance use and related problems’ [46]. The tool assesses for the abuse of several substances including, tobacco, alcohol, cocaine, sedatives, among others. It has been shown in several studies to be a valid and reliable (Cronbach’s α = 0.92 for alcohol, Cronbach’s α = 0.91 for cocaine, Cronbach’s α = 0.85 for cannabis, Cronbach’s α = 0.85 for opioids, Cronbach’s α = 0.87 for sedatives, and Cronbach’s α = 0.73 for tobacco) tool for identifying psychoactive substance use [46–48]. The scoring categorisation outlined by the ASSIST was used [46]. The ASSIST has been used before in Kenya [49].

### Statistical analysis

Descriptive statistics were used to examine the general distribution of the factors and outcomes by means, standard deviations and range for continuous variables and proportions for categorical variables. Cut off points for CESD-R, PHEEM, PSS, and MSPSS were used to determine the proportion of individuals in each sub-group. These were summarized in the form of tables. Independent sample t-test and one-way ANOVA was carried out for the bivariate analysis, in order to test for the difference in the means between the socio-demographic characteristics and CESD-R. Post-hoc analysis was then done on the significant variables in the ANOVA to find out where the differences occurred between the groups (see Additional file 2). One way ANOVA was also carried out between Medical specialty and MSPSS, PSS, PHEEM, and ASSIST. To assess the relationship between total depression score (CESD-R), PSS, PHEEM, MSPSS, and ASSIST, Pearson correlation was used. Significant independent variables in the bivariate analysis i.e. *p* < 0.05 were used to add independent variables in the multivariate analysis. Multivariate analysis was carried out to determine independent predictors of depression by regressing Total CESD-R scores to the significant socio-demographic variables, PSS, PHEEM, MSPSS, and substance use at the bivariate level using generalized linear model. The level of statistical significance was set at *p* < 0.05 (two- sided). We did not assess for normality because we used a sample size of 338 therefore violation of normality was not of much concern. All analyses were conducted using the Statistical Package for Social Sciences (SPSS) version 23.0.

## Results

Of the 338 respondents: 46.6% were female, the mean age was 31.8 years (SD = 3.07), 55.6% (*N* = 188) were married, 2.4% were separated and or divorced (see, Table 1). Majority of residents (*N* = 266, 78.7%) did not have any relatives with depression, 94% had never been diagnosed with depression before. One of the findings that points to sedentary lifestyle related issues that caught our attention was that most residents (*N* = 111, 32.9%) did not perform any physical exercise during the week, the second largest group of residents (26.4%) only exercised less than an hour a week. Most residents (74.4%) actively participated in leisurely activities/hobbies. On average residents only had 2.7 (SD = 2.18) days a week where they obtained between 7 and 9 h of sleep in a night. (see Table 1).

**Table 1:** Sociodemographics

Variable	N	Mean (SD)/Frequency (%)
Age, mean years (SD)	320	31.8 (− 3.07)
Gender, n (%)	337	
Female		157 (46.6)
Male		180 (53.4)
Religion, n (%)	335	
Catholic		81 (24.2)
Protestant		164 (49)
Muslim		57 (17)
Other		33 (9.9)
Medical specialty, n (%)	338	
Internal medicine		59 (17.5)
Pediatrics		51 (15.1)
Obstetrics and Gynecology		77 (22.8)
General surgery		47 (13.9)
ENT surgery		28 (8.3)
Cardiothoracic surgery		9 (2.7)
Anesthesia		38 (11.2)
Psychiatry		29 (8.6)
Relationship status, n (%)	338	
Single (never married)		90 (26.6)
Dating		52 (15.4)
Married		188 (55.6)
Separated/ Divorced		8 (2.4)
Exercise per week, n (%)	337	
0 h.		111 (32.9)
< 1 h		89 (26.4)
1–2.5 h		75 (22.3)
> 2.5 h		62 (18.4)
Type of exercise, n (%)	334	
Aerobic (jogging, walking,		150 (44.9)
Strength		27 (8.1)
Aerobic and strength		30 (9.0)
Other (specify)		16 (4.8)
None		111 (33.2)
Hobbies, n (%)	336	
Yes		250 (74.4)
No		86 (25.6)
Family member with depression, n (%)	338	
Yes		72 (21.3)
No		266 (78.7)
Previous diagnosis of depression, n (%)	336	
Yes		20 (6)
No		316 (94)
Illness other than depression, n (%)	337	
Yes		36 (10.7)
No		301 (89.3)
Number of children, n (%)	331	
None		155 (46.8)
One		84 (25.4)
Two		69 (20.8)
3 or more		23 (6.9)
Total monthly income in Kenyan Shillings, n (%)	332	
10,000 – 50,000		22 (6.6)
51,000 – 100,000		35 (10.5)
101,000 – 150,000		79 (23.8)
> 150,000		196 (59)
Days you had 7 to 9 h of sleep, mean number of days (range)	331	2.7 (0–7)

### Depression

The average score for depressive symptoms was 12.03 (SD = 12.26) out of a maximum score of 60. Out of all 338 residents 238 (70.4%) had mild to no depressive symptoms, 43 (12.7%) had moderate symptoms, and 57 (16.9%) had severe symptoms (see, Table 2). The dimensions of depression with the highest average scores were sleep (M = 2.23, SD = 2.28), and sadness (M = 2.16, SD = 2.46).

**Table 2:** Frequency distribution of CESD-R, MSPSS, PSS, PHEEM, and ASSIST

Variable	Category	Frequency (*N* = 338)	Percent %	Min	Max	Mean (SD)
Depression (CESD-R)	Mild/No depression	238	70.4	0	55	12.03 (12.26)
	Moderate	43	12.7			
	Severe	57	16.9			
Social Support (MSPSS)	Low support	16	4.7	1	7	5.57 (1.18)
	Moderate support	79	23.4			
	High support	242	71.8			
	***Missing***	***1***				
Perceived Stress (PSS)	Low stress	86	25.6	0	36	18.58 (6.89)
	Moderate stress	207	61.6			
	High perceived stress	43	12.8			
	***Missing***	***2***				
Educational Environment (PHEEM)	Very poor	18	5.3	13	156	83.82 (26.96)
	Plenty of problems	138	40.9			
	More positive than negative	156	46.3			
	Excellent	25	7.4			
	***Missing***	***1***				
Drug Use (ASSIST)		**Total (N)**	**Low risk**	**Moderate**	**High risk**	**Mean (SD)**
	**Drug**		**n (%)**	**n (%)**	**n (%)**	
	Tobacco	197	180 (91.4%)	15 (7.6%)	2 (1.0%)	1.56 (5.18)
	Alcohol	240	201 (83.8%)	32 (13.3%)	7 (2.9%)	5.47 (7.12)
	Cannabis	196	182 (92.9%)	14 (7.1%)	0 (0%)	0.76 (2.56)
	Cocaine	238	211 (88.7%)	27 (11.3%)	0 (0%)	0.52 (1.46)
	Amphetamine	176	173 (98.3%)	3 (1.7%)	0 (0%)	0.19 (1.45)
	Sedatives	179	172 (96.1%)	6 (3.4%)	1 (0.6%)	0.56 (3.19)
	Hallucinogens	176	174 (98.9%)	2 (1.1%)	0 (0%)	0.14 (1.09)
	Opioids	179	176 (98.3%)	3 (1.7%)	0 (0%)	0.17 (1.65)
	Total substance abuse	337				9.11 (13.74)

### Perceived stress

The mean score was 18.58 (SD = 6.89). A total of 336 residents completed the PSS, out of these: 86 (25.6%) had low stress, 207 (61.6%) had moderate stress, and 43 (12.8%) had high stress (see, Table 2). There was no significant difference in PSS scores between the different departments (see, Table 5).

### Perceived social support

The average score for the 337 residents that completed the MSPSS questionnaire was 5.57 (SD = 1.18). Most residents 242 (71.8%) experienced high levels of social support, 79 (23.4%) had moderate social support, and only 16 (4.7%) had low social support (see, Table 2). The “Friends” subscale was the lowest rated dimension for all specialties. There was no significant difference between MSPSS scores between different departments (see, Table 5).

### Hospital educational environment

The average PHEEM score was 83.82 (SD = 26.96). Out of 337 residents that completed this questionnaire: 18 (5.3%) rated their educational environment as very poor, 138 (40.9%) as having plenty of problems, 156 (46.3%) as being more positive than negative, and 25 (7.4%) as being excellent (see, Table 2). There was no significant difference between PHEEM scores between different departments (see, Table 5).

### Substance use

There was no significant difference in ASSIST scores between different departments. Out of 197 respondents: 180 (91.4%) were low risk, 15 (7.6%) were moderate, and 2 (1.0%) were high risk of suffering health and other problems due to tobacco use. Out of 240 respondents to questions on alcohol: Most (83.8%) were low risk, 32 (13.3%) were moderate, and 7 (2.9%) were high risk. Of the 238 respondents that responded to cocaine use: 211 (88.7%) were low, 27 (11.3%) moderate, none were high risk (see, Table 2).

### Bivariate associations between depression and other mental health measures

Our results showed that depression was significantly associated with perceived stress *(r = 0.618),* substance use *(r = 0.186),* social support *(r = − 0.443),* and educational environment *(r = − 0.304)* (see, Table 3). There were also significant associations between all of the measures i.e., perceived social support, perceived stress, substance use, and educational environment (see, Table 3). Depression was also significantly associated with social and personal demographic characteristics such as female gender, and less than 1 hour of exercise a week (see, Table 4).

**Table 3:** Correlational matrix between CESD-R, PSS, MSPSS, PHEEM, and ASSIST scores

Pearson correlation	CESD-R	PSS	MSPSS	PHEEM	ASSIST
CESD-R	1				
PSS	0.618**	1			
MSPSS	−0.443**	−0.302**	1		
PHEEM	−0.304**	−0.340**	0.301**	1	
ASSIST	0.186**	0.152**	−0.157**	− 0.118*	1

** Correlation is significant at the 0.01 level (2-tailed)

* Correlation is significant at the 0.05 level (2-tailed)

*CESD-R* Centers for Epidemiologic Studies Depression Scale Revised

*MSPSS* Multidimensional Scale of Perceived Social Support

*PHEEM* Postgraduate Hospital Educational Environment Measure

*PSS* Perceived Stress Scale

*ASSIST* Alcohol, Smoking and Substance Involvement Screening Test

**Table 4:** Multivariate and bivariate associations between depression and other mental health measures

Multivariate analysis					
Parameter		**β**	**95% Wald Confidence Interval**		**Sig.**
Variable	**Category**		**Lower**	**Upper**	
Gender	Female	2.93	0.844	5.019	0.006*
	Male	Ref			
Medical specialty	Internal medicine	3.24	−0.679	7.158	0.105
	Pediatrics	5.57	1.508	9.624	0.007*
	Obstetrics and Gynecology	4.27	0.574	7.958	0.024*
	General surgery	1.82	−2.275	5.917	0.384
	ENT surgery	4.61	0.169	9.040	0.042*
	Cardiothoracic	0.69	−5.923	7.320	0.836
	Anesthesia	3.79	−0.348	7.935	0.073
	Psychiatry	Ref			
Hours of exercise per week	0 h.	1.25	−1.532	4.025	0.379
	< 1 h	−1.23	−4.042	1.579	0.391
	1–2.5 h	−1.05	−3.895	1.792	0.469
	> 2.5 h	Ref			
Hobbies	Yes	− 1.84	−3.998	0.325	0.096
	No	Ref			
Family depression	Yes	1.85	−0.408	4.117	0.108
	No	Ref			
Previous diagnosis of depression	Yes	10.07	6.136	14.007	< 0.001*
	No	Ref			
Current illness other than depression	Yes	3.06	0.095	6.023	0.043*
	No	Ref			
Total household income per month in Kenyan Shillings	10,000 – 50,000	4.54	0.878	8.202	0.015*
	51,000 – 100,000	2.04	−1.075	5.153	0.199
	101,000 – 150,000	3.01	0.792	5.232	0.008*
	> 150,000	Ref			
Sleep hours		−0.68	−1.125	−0.242	0.002*
Total PHEEM		−0.01	−0.05	0.026	0.529
Total PSS		0.71	0.556	0.863	< 0.001*
Total MSPSS		−2.19	−3.023	−1.357	< 0.001*
Total ASSIST		0.07	−0.001	0.137	0.053
Bivariate Analysis					
Variable	**Category**	**β**	**95% Confidence Interval**		
			**Lower**	**Upper**	**Sig.**
Gender	Female	4.48	1.91	7.05	0.001*
	Male	Ref.			
Hobbies	Yes	−4.61	−7.56	−1.65	0.002*
	No	Ref.			
Family depression	Yes	6.43	3.31	9.54	< 0.001*
	No	Ref.			
Previous diagnosis of depression	Yes	15.08	9.78	20.38	< 0.001*
	No	Ref.			
Current illness other than depression	Yes	7.40	3.24	11.56	< 0.001*
	No	Ref.			
Medical specialty	Internal medicine	−0.17	−5.47	5.14	0.951
	Paediatrics	5.19	−0.25	10.63	0.061
	Obstetrics and Gynaecology	3.07	−2.03	8.17	0.238
	General surgery	−2.15	−7.67	3.38	0.446
	ENT surgery	2.67	−3.53	8.87	0.398
	Cardiothoracic surgery	−7.05	−15.98	1.88	0.122
	Anaesthesia	0.09	−5.68	5.86	0.975
	Psychiatry	Ref.			
Hours of exercise per week	0 h	5.58	1.84	9.32	0.003*
	< 1 h	0.92	−2.99	4.82	0.645
	1–2.5 h	2.03	−2.02	6.08	0.326
	> 2.5 h	Ref.			
Total household income per month in Kenyan Shillings	10,000 – 50,000	8.27	2.93	13.61	0.002*
	51,000 – 100,000	−0.09	−4.45	4.27	0.968
	101,000 – 150,000	3.07	−0.09	6.24	0.057
	> 150,000	Ref.			
Sleep hours		−1.29	−1.88	−0.71	< 0.001*
Total PHEEM		−0.14	−0.18	− 0.09	< 0.001*
Total PSS		1.10	0.95	1.25	< 0.001*
Total MSPSS		−4.58	−5.57	−3.59	< 0.001*
Total ASSIST		0.17	0.07	0.26	0.001*

*Correlation is significant at the 0.05 level

### Multivariate analysis of depression

There were significant associations with depression and sociodemographic factors such as being a female (β = 2.932, *p = 0.006*), and average income of less than KSH 100,000 to KSH 150,000 (β = 4.54, *p = 0.015*) (see, Table 4). We found that residents in the following departments: paediatrics department (β = 5.566, *p = 0.007*), obstetrics/gynaecology department (β = 4.266, *p* = 0.024), and the ENT surgery department (β = 4.605*, p = 0.042*) were found to have higher scores of depression than others. Being diagnosed with another illness (β = 3.059, *p = 0.043*), was also associated with higher scores of depression. (see, Table 4).

Amongst other cofactors of mental disorders/distress, we found that depression was also strongly associated with: fewer hours of sleep (β = − 0.683, *p = 0.002*), perceived stress (β = 0.709, *p < 0.001*), and perceived social support (β = − 2.19, *p < 0.001*). Depression was not significantly associated with substance use (β = 0.068, *p = 0.053*), and educational environment (β = − 0.012, *p = 0.529*) (see, Table 4).

### Post hoc analysis between medical specialties

There was no significant difference between medical specialties and MSPSS, PHEEM, PSS, and ASSIST scores (see, Table 5). However, there was a significant difference between medical specialties’ depression scores (*p* = 0.02).

**Table 5:** One way ANOVA between medical specialty and CESDR, PHEEM, PSS, MSPSS, and ASSIST

Variable	df	Mean Square	F	Sig.
CESDR	7	354.246	2.427	*0.02
PHEEM	7	1338.687	1.875	0.073
PSS	7	67.492	1.438	0.189
MSPSS	7	1.139	0.808	0.581
ASSIST	7	132.431	0.697	0.675

*Significance at 0.05 level

## Discussion

We found that most residents 238 (70.4%) had no to mild depressive symptoms, severe symptoms were present in 57 (16.9%) residents, with 43 (12.7) reporting moderate depressive symptoms. Most residents had high perceived social support and most had moderate stress. We found significant associations between depression and less sleep, high perceived stress, and low perceived social support.

The prevalence of depressive symptoms we found is similar to previous studies reported globally [50, 51]. Our findings contrast with those of Raviola et al. [6] who conducted structured DSM IV interviews with 50 residents at the same hospital in Kenya in 2002. They sampled third and fourth year residents from general surgery, internal medicine, paediatrics, and obstetrics and gynaecology. Their study found that 48% of residents met the criteria for major depression. This difference in prevalence estimates can be explained by differences in methodology between the two studies: with our study targeting more residents (338 vs. 50) from more specialties (8 different specialties in our study vs. 4 in Raviola’s), from all years of study, using a different instrument to measure depression than theirs (our study used the CESD-R self administered questionnaire while Raviola’s used structured DSM IV interviews which is a more individualized, clinician administered assessment). Our results can also be explained by changes in the work environment, and pay, as well as the drastic changes that have occurred in the country and hospital in the 17 years following Raviola’s study. Compared to 17 years ago, residency programs now have higher pay (however not all residents are enrolled in programs that pay them), have a higher number of residents which may ease the workload, and residency programs do not discourage residents from working as locum doctors in other hospitals. Anecdotally, the older programs had strict hierarchical structures with limited interaction between residents and consultants which is no longer the case for most consultants and residents. Another interesting comparison albeit with a study that looked at a different type of population is with Othieno et al’s study [8] that used the CESD to measure depression among university students in Kenya in 2014. Their study found that most students had scores in the first two, less severe categories of depression i.e. mild (41.3%) and moderate (35.7%); ours however had a higher proportion of scores in the two extreme categories of depression i.e. mild (70.4%) and severe (16.9%).

Compared to the general population the prevalence of depression we found is significant given that the previous estimates for common mental disorders in the general population through household surveys were around 10.8% with 0.7% point prevalence for depressive disorders [52, 53]. In a follow up study, over the last decade 2004–13, the prevalence of depression for men dropped from 10.9 to 3.8% (*P* = 0.001) and the prevalence for women increased from 10.8 to 17.5% (*p* = 0.001) in the general population [54]. Our findings could be explained by the stressful, mentally, and physically demanding nature of medical residency programs. To name a few of the key challenges residents face: residents often are unable to obtain sufficient sleep, they have to deal with difficult clinical cases, have to read and understand large volumes of information, and at times fail to spend time with their loved ones due to the demanding nature of their training. The effects of these challenges may be exacerbated by a sub-optimal organisational structure with regards to promotion of good mental health, resident welfare, and self care practices. Some residency programs may have formal structures to address resident counselling, however they may be underutilised for a variety of reasons, including limited operating hours for clinics, resident unawareness, and fear of appearing ‘weak’ by residents. Other factors such as poor coping skills and the easy access to substances of abuse serve only to compound these problems among residents. The residents in our study reported high levels of social support despite these challenging experiences. The availabilility of social support likely helps them cope with their stresses and therefore prevents or reduces depressive symptoms. It is perhaps also known to their family and friends that their everyday life experiences are difficult and stressful. Anecdotally, some residents suggested that senior residents and consultants that are friendly and supportive have on many occasions helped them cope with their challenges; and in the absence of supportive lecturers or senior residents the professional learning experiences become difficult.

In our study, the bivariate analysis revealed that stress, substance use, low social support, and a poor educational environment may be risk factors for depression., Each construct of interest for our cohort (e.g. depression, educational environment, social support, etc) was significantly correlated to the other. Our multivariate analysis results show that high levels of perceived social support, adequate sleep, and high income may be protective against depression. While stress, substance use, female gender, current comorbid illnesses, and previous diagnoses of depression may be risk factors for depression. An alternative explanation is that those experiencing more depressive symptoms may be more likely to perceive less social support, have less sleep, experience more stress, and use more substances as a result of their depressive symptoms. However it is important to note that this is a cross-sectional survey that used self-assessment tools therefore causality cannot be determined.

Perceived stress scores (both means and proportions) were similar to the levels found in other studies in LMICs [14, 55, 56]. The average score we found was slightly lower than that found in a recent study in the same population in Kenya, we however found a larger proportion of residents in the moderate and high perceived stress category compared to the other study [11]. Given that moderate and high stress are prevalent in our study the possibility of the negative effects of stress are therefore more likely in this cohort. This may imply that the residents we studied are more likely to make medication errors, experience burnout, and have reduced empathy towards patients [12, 13].

The average PHEEM score in our study was higher than previous studies [29, 57] indicating more positive views about the learning environment in our study, and this finding was similar to others from South Africa, Saudi Arabia and Morocco [58–60]. Almost half of the residents (46.3%) found their educational environment to be more positive than negative, and 40.9% found it more negative than positive. This indicates that the majority of residents feel that improvements can be made to their respective learning environments, with only 5.3, and 7.4% rating their educational environments as being very poor, and excellent respectively. Our bivariate analysis showed a significant negative correlation between depression and educational environment (*r* = − 0.304). This suggests that a positive perception of a residents’ educational environment may be protective against depression. Additionally, residents with fewer depressive symptoms may view their educational environment more favourably. We conjecture that there may be a pattern in residents towards using either an internal or external locus of control with regards to their hospital and training environment appraisal. Residents who may be internalizing distress and may be more prone to experience depressive symptoms, and those who externalize and think hospital environment is the cause of their distress might experience fewer to no depression.

A large majority of residents 242 (71.8%) perceived high levels of social support, with most support obtained from significant others and family, and the least support from friends. We found that social support was significantly negatively correlated with depression. This indicates that higher levels of social support may be protective against depression. It has been well-noted that good social support is an effect modifier when it comes to mental and physical health [61]. Social support is a multidimensional concept assessed across several domains (family, friends, and significant other) hypothesised to protect mental health both directly through the benefits of social relationships and indirectly as a buffer against stressful circumstances [62]. Thus the high levels of social support we found may explain the relatively low scores for depression in our study.

Alcohol was the most widely used substance with 2.9% of those who used it being at high risk of suffering from ill health and other negative effects due to its use. The majority (83.8%) were at low risk due to alcohol use. Tobacco was the next most commonly used substance with only 1% being at high risk, and the majority (91.4%) at low risk which is lower than previous studies in Africa (however they used different instruments to assess smoking prevalence) [63]. Only one resident was classified as high risk for sedative use. No residents were classified as high risk due to cannabis, opioids, cocaine, amphetamines, and hallucinogens. However 11.3% were at moderate risk due to cocaine use, and 7.1% due to cannabis, which represents a combined total of 41 residents. When considering these results it is important to note that the proportions given are for the residents who answered those specific items in the questionnaires, (not all 338 residents we sampled answered those specific questions). More specifically only 238 residents answered the question on cocaine, and only 196 answered the question on cannabis. This however, worryingly indicates potentially more common usage of cocaine and marijuana amongst our cohort compared to the general population in Kenya where only 1.0% use cannabis and less than 0.1% use cocaine [64]. When compared to university students in Kenya our results show less frequent use of cannabis with 8% of university students using cannabis [47]. The instrument we used to assess substance use did not collect information regarding the specific type of opioid used therefore we are unable to identify specific drugs. However no resident reported having ever used injectable drugs therefore eliminating the possibility of intravenous drugs like heroin being one of the opioids used. In a study on US medical residents, it was found that residents overall have lower rates of substance use than their age peers in society. Yet resident substance use patterns do differ by specialty and this study found emergency medicine and psychiatry residents have higher rates of use [65]. A survey on health care workers in Kenya used ASSIST and found that Reported lifetime use was 35.8% for alcohol, 23.5% for tobacco, 9.3% for cannabis, 9.3% for sedatives, 8.8% for cocaine, 6.4% for amphetamine-like stimulants, 5.4% for hallucinogens, 3.4% for inhalants, and 3.9% for opioids and suggests that the substance use in HCWs may be higher than the general population [66]. Our population is a unique group of medical doctors under training so their substance use patterns and risk factors including speciality associations need to be further explored.

We cannot claim to unravel causal relationships due to the nature of our study design. We used self- administered questionnaires which may allow residents to under report the severity of their symptoms due to the stigma associated with mental illness. Residents may not have openly filled in the questionnaires because they may not have seen an apparent benefit to completing the survey. There was also the possibility of residents filling in the questionnaires incorrectly.. Residents were encouraged to be forthright to prevent them giving false responses, however it was impossible to ensure and assess the truthfulness of responses.

There may also be biases around underreporting distress, use of harmful substances is often underreported in medical settings and wanting to look fit to maintain professional credence and avoid mental ill-health stigma are barriers to expressing the need for greater support. The cardiothoracic surgery department was small in size with only 9 residents being sampled after calculating the sample size using proportionate sampling (the department has a total of 14 residents). This may mean that results in our study from that specialty may not be generalisable to other cardiothoracic departments due to the small sample size in our study.

Given that we did not control for differences innate to different specialties, it may not be appropriate to compare different departments/specialties in our study to each other. The residents we sampled were in an urban setting therefore our results may not reflect the condition of doctors and residents in rural or remote settings which we anticipate may be further worse due to material and human resource constraints. In order to maintain anonymity, we were unable to contact residents who did not provide their contact details (which was optional), unfortunately those with more severe scores for stress, depression, and substance use did not provide any contact information so we were not able to offer them any assistance.

To our knowledge this is one of the few studies that have looked at combined depressive symptoms, social support, educational environment, and substance use in a structured mental health survey among residents (doctors in training) in East Africa. We sampled a large proportion of residents in one of the largest teaching hospitals in East Africa, we sampled residents from a wide range of surgical and non-surgical specialties. A fact that enhances the generalisability of our results as well as gives a detailed insight into the mental health of residents and by extension doctors in Kenya and sub-Saharan Africa. We also included residents from all years of study and therefore different age groups so that our results paint a better picture of residency as a whole and not of specific levels of study and age groups.

A key strength of our study is that we have also stratified and analysed results according to different departments which allows us to have a better understanding of the different specialties. This is important because each specialty has factors that are unique to them, not to mention other factors that are unique to individual programs within the same university.

To provide as global an understanding of residents’ mental health as possible we included validated questionnaires on a wide variety of concepts that affect mental health negatively i.e. depression, stress, substance use; and positively i.e. social support, and educational environment. We included questions on residents’ personal lives such as income, sleep, relationship status etc. to try to identify factors outside of the hospital that might play a role in their mental health.

We recommend that future studies on residents’ and doctors’ mental health should be carried out in East and sub-Saharan Africa with similar tools so that a more complete understanding of the region is available. Prospective studies looking at the effect of perceived social support, depression, stress, substance use, and educational environment should be carried out in residents in order to describe causal relationships. The reasons for such high social support among residents in our study should be further explored in order to understand why it is high and how it can be maintained and replicated in similar settings with lower levels of support.

Future interventions to prevent depression among residents should aim to strengthen the protective factors we found and eliminate/reduce the risk factors. We recommend a departmental approach to any interventions owing to the differences in the learning environments, lifestyles, and demands that are unique to each specialty and department. Attention should be given to the social determinants of health unique to residents to further augment mental health among them. We suggest measures such as easily accessible counselling services as well as designated rooms/areas for residents to meet and discuss the challenges that they face together. We also recommend strengthening mentorship by senior residents and consultants to help guide their juniors. Efforts should be made to understand the pattern of substance use among residents and to reduce substance use, this could be achieved through programs to raise awareness as well as the provision of counselling.

It is important to monitor and understand residents’ mental health given the increased risk they face of suffering from mental illness and the important role that they play in patient care. Preventing depression and poor mental health among residents may not just benefit the residents but may also reduce the numerous negative effects (of mental illness in doctors) on patient care that have been demonstrated in previous studies.

## Conclusions

Overall, our findings indicate that residents have moderate levels of depressive symptoms and stress, are at risk of abusing various substances and have high levels of social support. Efforts should be made to understand and maintain the high levels of social support, reduce the prevalence of depressive symptoms, and stress; and reduce the risk of substance abuse among residents in Kenya.

## Supplementary Information

**Additional file 1.** Sociodemographic questionnaire. The file contains the sociodemographic questionnaire that was used in our study in its entirety.

**Additional file 2: Supplementary Table 1.** Post Hoc ANOVA of Significant Variables.

## Data Availability

The datasets generated and/or analysed during the current study are not publicly available because no permission has been obtained from participants and the hospital/university (where the study was conducted) to publish datasets publicly. The authors also intend to further explore the data to generate more insights/findings and publish those new insights/findings. The data are available from the corresponding author on reasonable request.

## Abbreviations

DALYs: Disability Adjusted Life Years
ENT: Ear Nose Throat
KNH: Kenyatta National Hospital
DSM: Diagnostic and Statistical Manual of Mental Disorders
WHO: World Health Organisation
CESD-R: Centers for Epidemiologic Studies Depression Scale Revised
MSPSS: Multidimensional Scale of Perceived Social Support
PHEEM: Postgraduate Hospital Educational Environment Measure
PSS: Perceived Stress Scale
ASSIST: Alcohol, Smoking and Substance Involvement Screening Test
