# Supplementary material for: Depression and its associated factors: perceived stress, social support, substance use and related sociodemographic risk factors in medical school residents in Nairobi, Kenya
Source: BMC Psychiatry. 2021 Sep 8;21:444. doi: 10.1186/s12888-021-03439-0 (PMC8425003; doi:10.1186/s12888-021-03439-0)
Supplement: Supplementary file 1 — Additional file 1. Sociodemographic questionnaire. The file contains the sociodemographic questionnaire that was used in our study in its entirety. [file 12888_2021_3439_MOESM1_ESM.docx]

**Depression and Its Associated Factors: Perceived Stress, Social Support, Substance Use and Related Sociodemographic Risk Factors in Medical School Residents in Nairobi, Kenya**

**Author Affiliations**

Sayed Shah Nur Hussein Shah (corresponding author) (undergraduate 6^th^ year medical student),

School of Medicine,

University of Nairobi,

P.O. BOX 59-00621, Nairobi, Kenya,

Email: [shahnur2500@gmail.com](mailto:shahnur2500@gmail.com)

Ahmed Laving,

Consultant paediatrician, Kenyatta National Hospital

Senior lecturer, Department of Paediatrics and Child Health,

University of Nairobi,

Email: [arlaving@yahoo.com](mailto:arlaving@yahoo.com)

Violet Caroline Okech-Helu

Consultant psychiatrist, Department of Mental Health

Kenyatta National Hospital,

Email: [okechviolet25@gmail.com](mailto:okechviolet25@gmail.com)

Dr. Manasi Kumar, PhD

Department of Psychiatry

University of Nairobi

Nairobi Kenya 00100 (47074)

Email: [manni_3in@hotmail.com](mailto:manni_3in@hotmail.com)

## Socio-demographic questionnaire

| **SECTION 1** | |
| --- | --- |
| Date of interview (DD/MM/YY) |  |
| Interviewers’ name |  |
| Informed consent obtained (1=Yes 0=No) |  |
| Start time (Hr:Min) |  |
| End time (Hr:Min) |  |
| Total time (Hr:Min) |  |

| **SECTION 2** | | | |
| --- | --- | --- | --- |
| **No.** | **QUESTION** | **RESPONSE** | **CODE** |
| **1** | Age | Number |  |
| **2** | Sex | Female = 1  Male = 2 |  |
| **3** | Religion | 1. Catholic  2. Protestant  3. Muslim  4. Hindu  5. Atheist  6. Other (specify) |  |
| **4** | Specialty | 1. Internal medicine  2. Pediatrics  3. Obstetrics & Gynecology  4. General Surgery  5. ENT Surgery  6. Cardiothoracic Surgery  7. Anesthesia  8. Psychiatry |  |
| **5** | How many years does your residency program take? | Number |  |
| **6** | Current year of residency program | Number (1,2,3etc) |  |
| **7** | Relationship status | 1. Single (never married)  2. Dating  3. Married  4. Separated  5. Divorced  6. Widowed |  |
| **8** | Number of children | Number |  |
| **9** | How many hours a week do you exercise? | 1. 0hrs  2. <1hr  3. 1-2.5hrs  4. 2.5-5hrs  5. >5hrs |  |
| **10** | What type of exercise do you mainly perform? | 1. Aerobic(jogging, walking, zumba, etc)  2. Strength(weightlifting, resistance training, etc)  3. Aerobic and strength  4. Other (specify)  5. None |  |
| **11** | Do you have hobbies that you take part in? | 1. Yes  2. No |  |
| **12** | Has anyone in your family ever been diagnosed with depression? | 1. Yes  2. No |  |
| **13** | Have you ever been diagnosed with depression? | 1. Yes  2. No |  |
| **14** | Do you have any illness that is not depression? | 1.Mental illness other than depression(specify)  2.Other(specify)  3.No |  |
| **15** | If **yes** to **Q13**. Are you taking any medication for depression? (if yes, then specify) | 1.Yes(specify)  2.No |  |
| **16** | If **yes** to **Q13**. Are you undergoing any form of treatment other than medication for depression? Eg counselling (if yes, then specify) | 1.Yes(specify)  2.No |  |
| **17** | If **yes** to **Q14**. Are you undergoing any treatment for the stated illness? (if yes, then specify) | 1.Yes(specify)  2.No |  |
| **18** | In the past week (7days), how many days have you had between 7 and 9 hours of sleep? | Number (between 0 and 7) |  |
| **19** | Total household income per month in Kenyan Shillings | 1. 10,000 – 30,000  2. 31,000 – 50,000  3. 51,000 – 100,000  4. 101,000 – 150,000  5. >150,000 |  |
