# Supplementary material for: Depression and its associated factors: perceived stress, social support, substance use and related sociodemographic risk factors in medical school residents in Nairobi, Kenya
Source: BMC Psychiatry. 2021 Sep 8;21:444. doi: 10.1186/s12888-021-03439-0 (PMC8425003; doi:10.1186/s12888-021-03439-0)
Supplement: Supplementary file 2 — Additional file 2: Supplementary Table 1. Post Hoc ANOVA of Significant Variables. [file 12888_2021_3439_MOESM2_ESM.docx]

**Depression and Its Associated Factors: Perceived Stress, Social Support, Substance Use and Related Sociodemographic Risk Factors in Medical School Residents in Nairobi, Kenya**

**Author Affiliations**

Sayed Shah Nur Hussein Shah (corresponding author) (undergraduate 6^th^ year medical student),

School of Medicine,

University of Nairobi,

P.O. BOX 59-00621, Nairobi, Kenya,

Email: [shahnur2500@gmail.com](mailto:shahnur2500@gmail.com)

Ahmed Laving,

Consultant paediatrician, Kenyatta National Hospital

Senior lecturer, Department of Paediatrics and Child Health,

University of Nairobi,

Email: [arlaving@yahoo.com](mailto:arlaving@yahoo.com)

Violet Caroline Okech-Helu

Consultant psychiatrist, Department of Mental Health

Kenyatta National Hospital,

Email: [okechviolet25@gmail.com](mailto:okechviolet25@gmail.com)

Dr. Manasi Kumar, PhD

Department of Psychiatry

University of Nairobi

Nairobi Kenya 00100 (47074)

Email: [manni_3in@hotmail.com](mailto:manni_3in@hotmail.com)

**Supplementary Table 1: Post Hoc ANOVA of Significant Variables**

| Variable( I) | Variable (J) | Mean Difference (I-J) | Significance | 95% Confidence Interval | |
| --- | --- | --- | --- | --- | --- |
|  | | | | Lower Bound | Upper Bound |
| Specialty (I) | Specialty (J) |  |  |  |  |
| Internal medicine | Pediatrics | -5.359* | **0.021*** | -9.9 | -0.81 |
|  | Obstetrics & Gyn. | -3.235 | 0.123 | -7.35 | 0.88 |
|  | General surgery | 1.98 | 0.402 | -2.67 | 6.63 |
|  | ENT surgery | -2.839 | 0.307 | -8.29 | 2.62 |
|  | Cardiothoracic surgery | 6.883 | 0.112 | -1.62 | 15.39 |
|  | Anesthesia | -0.26 | 0.918 | -5.2 | 4.68 |
|  | Psychiatry | -0.167 | 0.952 | -5.56 | 5.22 |
| Pediatrics | Obstetrics & Gyn. | 2.124 | 0.331 | -2.17 | 6.41 |
|  | General surgery | 7.339* | **0.003*** | 2.53 | 12.14 |
|  | ENT surgery | 2.52 | 0.376 | -3.07 | 8.11 |
|  | Cardiothoracic surgery | 12.242* | **0.005*** | 3.65 | 20.84 |
|  | Anesthesia | 5.099* | **0.05*** | 0.01 | 10.19 |
|  | Psychiatry | 5.192 | 0.066 | -0.34 | 10.72 |
| Obstetrics & Gyn. | General surgery | 5.215* | **0.02*** | 0.82 | 9.61 |
|  | ENT surgery | 0.396 | 0.882 | -4.85 | 5.64 |
|  | Cardiothoracic surgery | 10.118* | **0.018*** | 1.75 | 18.49 |
|  | Anesthesia | 2.975 | 0.215 | -1.74 | 7.69 |
|  | Psychiatry | 3.069 | 0.245 | -2.11 | 8.25 |
| General surgery | ENT surgery | -4.819 | 0.096 | -10.49 | 0.85 |
|  | Cardiothoracic surgery | 4.903 | 0.266 | -3.74 | 13.55 |
|  | Anesthesia | -2.24 | 0.396 | -7.43 | 2.94 |
|  | Psychiatry | -2.147 | 0.452 | -7.76 | 3.47 |
| ENT surgery | Cardiothoracic surgery | 9.722* | **0.036*** | 0.61 | 18.83 |
|  | Anesthesia | 2.579 | 0.392 | -3.34 | 8.5 |
|  | Psychiatry | 2.672 | 0.404 | -3.62 | 8.97 |
| Cardiothoracic surgery | Anesthesia | -7.143 | 0.112 | -15.95 | 1.67 |
|  | Psychiatry | -7.05 | 0.127 | -16.12 | 2.02 |
| Anesthesia | Psychiatry | 0.093 | 0.975 | -5.77 | 5.95 |
|  |  |  |  |  |  |
| (I)Exercise | (J)Exercise |  |  |  |  |
| 0 hrs. | < 1hr | 4.659* | **0.007*** | 1.27 | 8.05 |
|  | 1-2.5hrs | 3.548 | 0.051 | -0.01 | 7.11 |
|  | >2.5hrs | 5.576* | **0.004*** | 1.8 | 9.35 |
| < 1hr | 1-2.5hrs | -1.111 | 0.559 | -4.84 | 2.62 |
|  | >2.5hrs | 0.917 | 0.647 | -3.02 | 4.86 |
| 1-2.5hrs | >2.5hrs | 2.028 | 0.33 | -2.06 | 6.12 |
|  |  |  |  |  |  |
| (I) type of exercise | (J) type of exercise |  |  |  |  |
| Aerobics | Strength | -3.027 | 0.234 | -8.02 | 1.97 |
|  | Aerobic and strength | -0.56 | 0.818 | -5.34 | 4.22 |
|  | Other (specify) | 2.619 | 0.413 | -3.67 | 8.9 |
|  | None | -4.801* | **0.002*** | -7.79 | -1.81 |
| Strength | Aerobic and strength | 2.467 | 0.445 | -3.87 | 8.81 |
|  | Other (specify) | 5.646 | 0.142 | -1.89 | 13.19 |
|  | None | -1.775 | 0.496 | -6.9 | 3.35 |
| Aerobic and strength | Other (specify) | 3.179 | 0.398 | -4.22 | 10.58 |
|  | None | -4.241 | 0.091 | -9.16 | 0.68 |
| Other (specify) | None | -7.421* | **0.023*** | -13.81 | -1.03 |
|  |  |  |  |  |  |
| (I) Income | (J) Income |  |  |  |  |
| 10,000 – 50,000 | 51,000 – 100,000 | 8.360* | **0.012*** | 1.83 | 14.89 |
|  | 101,000 – 150,000 | 5.197 | 0.078 | -0.59 | 10.98 |
|  | >150,000 | 8.270* | **0.003*** | 2.88 | 13.66 |
| 51,000 – 100,000 | 101,000 – 150,000 | -3.162 | 0.202 | -8.03 | 1.71 |
|  | >150,000 | -0.09 | 0.968 | -4.49 | 4.31 |
| 101,000 – 150,000 | >150,000 | 3.073 | 0.06 | -0.12 | 6.27 |

*The mean difference is significant at the 0.05 level
